# Urinary free cortisol assessment by liquid chromatography tandem mass spectrometry: a case study of ion suppression due to unacquainted administration of piperacillin

**DOI:** 10.11613/BM.2017.031001

**Published:** 2017-10-15

**Authors:** Elisa Danese, Gian Luca Salvagno, Alessandra Guzzo, Samuele Scurati, Cristiano Fava, Giuseppe Lippi

**Affiliations:** 1 Clinical Biochemistry section, Department of Neurological, Biomedical and Movement Sciences, University of Verona, Italy; 2 DASP s.r.l., Gerenzano, Italy; 3 Unit of General Medicine and Hypertension, Department of Medicine, University of Verona, Italy

**Keywords:** urinary free cortisol, ion suppression, pre-analytical phase, piperacillin, mass spectrometry

## Abstract

**Introduction:**

Liquid chromatography coupled to atmospheric pressure ionization tandem mass spectrometry (LC-ESI-MS/MS) is currently considered the reference method for quantitative determination of urinary free cortisol (UFC). One of the major drawbacks of this measurement is a particular form of matrix effect, conventionally known as ion suppression.

**Materials and methods:**

We describe here the case of a 66-year-old-patient referred to the daily service of general medicine for intravenous antibiotic administration due to a generalized *Staphylococcus aureus* infection and for routine 24 hours UFC monitoring in the setting of glucocorticoid replacement therapy.

**Results:**

The observation of 10-fold decrease of internal standard of cortisol signal led us to hypothesize the presence of an ion suppression effect due to a co-eluting endogenous compound. Screening analysis of tandem mass spectrometry (MS/MS) spectra of the interfering molecule, along with *in vitro* confirmation analyses, were suggestive of the presence of high concentration of piperacillin. The problem was then easily solved with minor modifications of the chromatographic technique.

**Conclusions:**

According to our findings, antibiotic therapy with piperacillin/tazobactam should be regarded as an important interference in UFC assessment, which may potentially affect detection capability, precision and accuracy of this measurement. This case report emphasizes that accurate anamnesis and standardization of all phases of urine collection are essential aspects for preventing potential interference in laboratory testing.

## Introduction

According to current guidelines, urinary free cortisol (UFC) is one of the first-line biochemical test for screening endogenous Cushing syndrome (*1*). Due to its higher selectivity and specificity compared to conventional immunoassays, liquid chromatography - tandem mass spectrometry (LC-MS/MS) has become the reference technique for measuring UFC in clinical practice. Despite an incomparable selectivity, MS detection is not completely foolproof, especially when coupled with electrospray ionization (ESI) sources, the most widely used interfaces used for UFC assessment. One of the most important factors impairing the accurate performance of a LC-MS/MS technique is the ion suppression (IS)/enhancement effect, an actual expression of a wider phenomenon conventionally known as “matrix effect”. Ion suppression occurs when matrix components co-eluting with compound/s of interest impair the efficiency of analyte ionization, thus affecting detection capability, precision and accuracy of measurements (*2*).

Although many authors recently underscored the importance of understanding IS effect in clinical MS applications, only a limited number of validations of methods have been published to consistently address this issue. To date, studies reporting an evaluation of matrix effect in UFC assessment by LC-MS/MS failed to observe any IS in study samples (*3*, *4*).

We report here for the first time a case study of IS due to high concentration of piperacillin in the urine sample. This paradigmatic case of biological interference which may be prevented by an accurate collection of patient information prior to analysis, reinforces the concept that pre-analytical phase remains crucial for quality of the total testing process, even when using high performance techniques such as LC-MS/MS (*5*-*8*). Since piperacillin is commonly used as a first-choice antibiotic in combination with the beta-lactamase inhibitor tazobactam for treating moderate-to-severe infections in critical patients, we describe here the analytical challenge of, and a valid approach to overcome, pre-analytical issues in measurement of UFC, which might be a rather frequent event in clinical practice (*9*).

## Laboratory analysis

### Case description

We describe here the case of a 66-year-old female patient referred to the daily service of general medicine of the University Hospital of Verona (Italy), for intravenous antibiotic treatment of a generalized *Staphylococcus aureus* infection. The clinical history, based on a thoughtful anamnesis, revealed Addison disease occurred after surgical management of a Cushing’s syndrome. The patient also received hydrocortisone therapy for four years due to adrenal insufficiency.

During routine assessment, 24-hours urine samples were collected for monitoring the glucocorticoid replacement therapy (*10*). The 24 h urine samples were collected in a standard 24 h urine container, with no preservatives. Urine was then aliquoted and stored at - 20 °C until analysis. The method currently used for assessing UFC in our hospital laboratory was a competitive solid phase ^125^I radioimmunoassay (RIA; Cortisol RIA kit, Beckman Coulter, Brea, USA). The bound radioactivity was measured with COBRA II Gamma counter (Packard, Ramsey, USA). The results of UFC assessment with this method, along with other relevant laboratory tests ordered at the time of urine sample collection are shown in Table 1. The study protocol was approved by the University Hospital of Verona Institutional Review Board.

**Table 1:** Routine clinical chemistry values

**Parameter**	**Value**	**Unit**	**Reference value**
**C-reactive protein**	< 3	mg/L	< 5
**Haematocrit**	0.36	L/L	0.35-0.47
**Haemoglobin**	116	g/L	120-150
**Creatinine**	158	μmol/L	44-106
**eGFR (CKD-EPI)**	29	mL/min/1.73m^2^	> 60
**UFC**	690	nmol/L	41.3-220.7
	2484	nmol/24h	
UFC - urinary free cortisol. eGFR - estimated glomerular filtration rate. CKD-EPI - Chronic Kidney Disease Epidemiology Collaboration.			

### What happened?

Due to the extremely high UFC value, the urine sample was aliquoted immediately after analysis and then stored at - 20 °C. Aliquots (of 300 μL, each) were then used for validating the inter-assay variability of a new commercial CE-IVD LC-MS/MS method, which had been recently introduced in our laboratory for replacing the RIA technique. The assay was the MS urinary free cortisol/cortisone kit (ISBN-BSN, Castelleone, Italy), which requires single manual dilution, protein precipitation and contains a D4 isotope-labelled internal standard. The method was found to be linear up to 5000 nmol/l and is characterized by a limit of quantification (LOQ) of 6 nmol/L.

The LC-MS/MS analytical system was a Nexera X2 series UHPLC (Shimadzu, Kyoto, Japan) coupled with a 4500 MD triple quadrupole MS detector (Sciex, Milan, Italy).

Compared to the other specimens tested in the same analytical run, the case study sample displayed a 10-fold lower signal (in terms of area under the peak) of internal standard (D4-Cortisol). Even the cortisol signal and the corresponding quantitative UFC concentration were very low compared to those we would have expected from data obtained with RIA. The software integration allowed generating a predicted UFC concentration of 229 nmol/L. However, due to the high degree of IS, normalization of cortisol value by its internal standard was inherently inaccurate and the following quantification was hence unreliable. A series of experiments were then carried out for confirming the presence of IS, understanding the source of the problem, and ultimately overcoming the possible interference.

### Evaluation of IS

**Figure 1:**
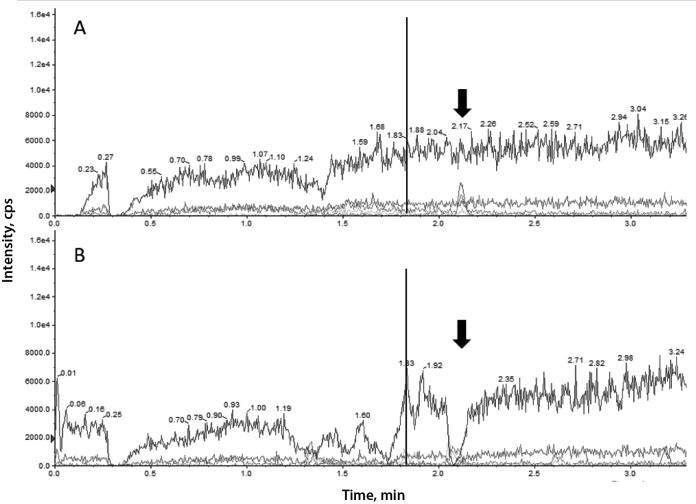
Post-column infusion experiment for evaluation of ion suppression affecting cortisol signal in a normal urine sample (A) and in the case-study sample (B). Black arrows indicate the elution time of cortisol. Vertical lines indicate the valve switch from the exhaust to the mass spectrometer. Cps - counts per second.

A post column infusion experiment was performed for assessing IS. A urine sample with low cortisol concentration (*i.e*. 13 nmol/L) and the case-study urine sample were injected while a cortisol solution (1 μmol/L) in 80:20 mobile phase A:B was infused into the mass spectrometer at a flow rate of 10 μL/min. As expected, a drop in the baseline signal occurring exactly in the elution time of cortisol was observed in the case sample but not in the other urine sample (Figure 1).

### Identification of interfering compound

The profile of the interfering compound was studied with a hybrid triple quadrupole/linear ion trap mass spectrometer (QTRAP 5500, Sciex, Milan, Italy) interfaced with a Nexera X2 series UHPLC (Shimadzu, Kyoto, Japan). General screening of interference was made by using the full-scan enhanced MS modality. Parent ions presenting with signal threshold > 100,000 counts per second (cps) were automatically fragmented to obtain the corresponding full-scan MS/MS spectra. Among all acquired compounds, those with retention time close to that of cortisol were analysed against the Analyst software (Sciex, Milan, Italy) MS/MS spectra database to provide tentative identification of compound. A match was finally found, which allowed identifying piperacillin as the interfering substance.

### Identity verification of interfering compound

With the aim of confirming piperacillin interference, the LC-MS/MS method was modified by adding the multiple reaction monitoring (MRM) m/z transitions of piperacillin. The subsequent re-analysis of case-study sample showed the presence of all set up transitions for both cortisol and piperacillin, thus confirming the presence of this drug in the study sample. An audit with the clinicians revealed that the patient was currently taking intravenous piperacillin/tazobactam treatment (2.25 g every 6 hours).

**Figure 2:**
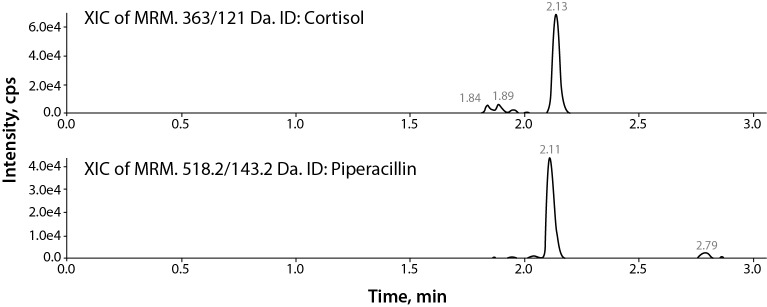
MRM chromatograms of cortisol and piperacillin. Representative chromatograms of a urine sample with UFC of 110 nmol/L spiked with piperacillin at a final concentration of 125 μg/mL. Cps - counts per second. XIC - Extracted ion chromatogram. MRM - multiple reaction monitoring.

With the aim of reproducing this phenomenon *in vitro*, scalar dilutions of the drug (piperacillin/tazobactam 4 g/0.5 g) were spiked into a urine sample (UFC: 110 nmol/L), to achieve final concentrations between 15.6-1000 μg/mL. Samples were then extracted according to the standard protocol and analysed with the modified method. As expected, the drug displayed an elution time very close to that of cortisol (Figure 2). Moreover, the sample spiked with a drug concentration of 1000 μg/mL displayed a signal for the second product very similar to that observed in the case-study sample (*i.e*., area of the m/z transition 518.2/160.1 of 20 and 17 million, respectively). The signals from the first product ion (m/z transition 518.2/143.2) were saturated instead in both samples.

**Figure 3:**
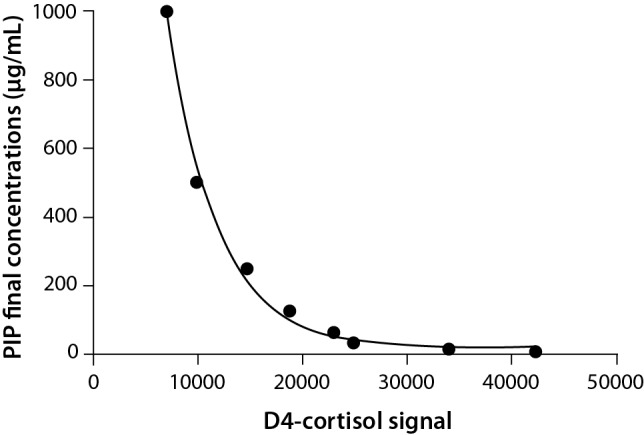
D4-cortisol signal suppression according to piperacillin increasing concentrations. Starting from a standard solution of 2000 μg/mL of piperacillin the follow scalar dilutions were spiked into an urine sample with UFC concentration of 110 nmol/L (1:2, 1:4, 1:8, 1:16, 1:32, 1:64). The curve was fitted by using a non-linear regression one-phase decay analysis and showed a correlation of 0.99. PIP – piperacillin.

As shown in Figure 3, the addition of piperacillin at final concentration of 1000 μg/mL in the urine sample induced an 80% signal decay compared to the sample without the spiked drug. By progressively diluting the piperacillin concentrations, the signal of the internal standard linearly increased, until reaching a recovery of 80% at a final concentration of 15.6 μg/mL.

## Solution

### Strategy implemented to remove IS due to piperacillin interference

**Figure 4:**
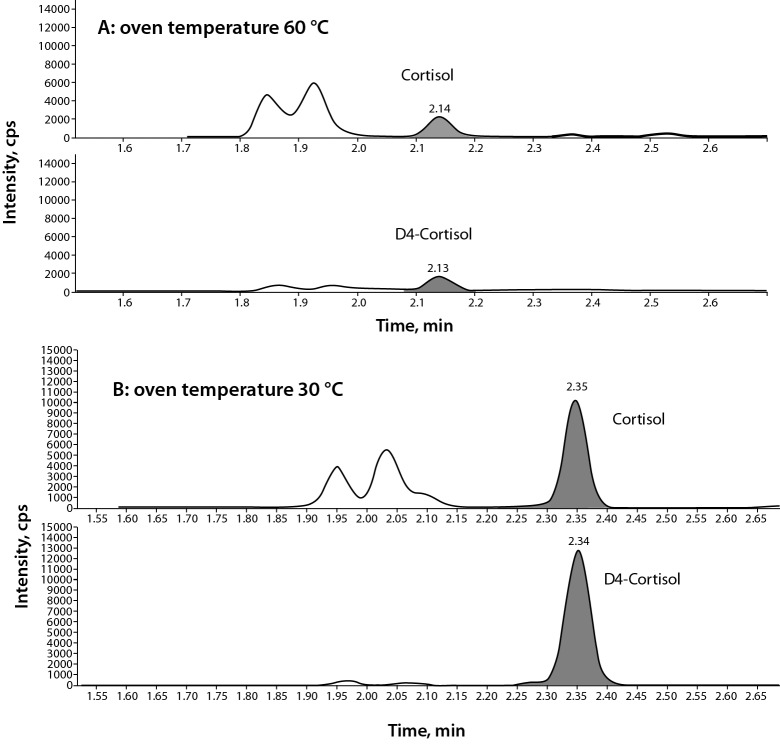
Cortisol and D4-cortisol signals before (A) and after (B) method modification. Cps - counts per second.

Among the many strategies available to eliminate or attenuate the matrix effect, we decided to improve retention and separation of co-eluting compound by re-optimizing the chromatographic conditions. As shown in Figure 4, the cortisol and the relative internal standard were found to be more retained by the C18 column after reducing the oven temperature from 60 °C to 30 °C. Consequently, their signals had no more signs of IS. The concentration of UFC could hence be calculated as 130 nmol/L after this minor modification.

## Discussion

Ion suppression is one of the most important challenges in MS analysis, so that is has been defined as the Achilles’ heel of quantitative LC–ESI-MS/MS analysis (*11*). This case report describes an IS effect attributable to piperacillin, seriously impairing the accurate assessment of UFC despite the use of an isotope labelled internal standard (which is usually enough to correct for the matrix effect). The initial quantification led to overestimating the real concentration of approximately 80% due to reduced detection capability of decreased UFC and internal standard signals. Notably, an even higher overestimation was noticed using the RIA, which was probably due to additional interference with this assay.

Although we did not performed a real quantification of the drug in the 24 hours urine sample, it is reasonable to assume, by comparing *ex vivo* and *in vitro* data, that 50-60% of the drug accumulated unmodified in urine (*i.e.* about 1 g/L) during the 24 hours collection. By assuming the most widely accepted linear pharmacokinetics profile and renal clearance of piperacillin, we can hence speculate that a IS effect caused by this drug might affect the UFC quantification, thus generating a signal reduction of up to 80% during infusion treatment, and up to 20% five hours after the last administration (*12*). To exclude this important pre-analytical interference, urine sample collection for UFC assessment should hence be started the day after the end of *i.v.* infusions. Even more importantly, accurate information about the pharmacological treatment should be collected and sent to the laboratory along with sample and prescription. Therefore, this case report further emphasizes the importance of a thoughtful collaboration between the clinics and the laboratory for identifying possible pre-analytical source of interference and preventing diagnostic errors.

The improvement of methods using internal standards seems also mandatory to identify this potential challenge, especially when measuring isolate samples. By normalizing analyte response signal, the internal standard may also serve to compensate for IS, thus overcoming biological interference. However, when the IS is so severe to jeopardize the correct identification of signals from both analyte and internal standard (as in this case), many strategies exist to overcome or reduce the problem, some of which may also be relatively simply as those herein described (*13*, *14*).

## What YOU should/can do in your laboratory to prevent such errors

Accurate assessment of urinary free cortisol by LC-MS/MS can be impaired by antibiotic therapy with piperacillin/tazobactam.
Matrix effect caused by the co-eluting drug can be recognized by implementing methods with isotope-labeled internal standard.
Potential interferences can be prevented by knowing patient’s pharmacological treatment.
By knowing pharmacokinetics of interfering drugs a correct timing for urine sample collection can be planned.
Once recognized, matrix effects can be resolved by re-optimizing the chromatographic conditions.
